# Assessment of Fatty Acid Concentrations Among Blood Matrices

**DOI:** 10.3390/metabo15070482

**Published:** 2025-07-17

**Authors:** Ysphaneendra Mallimoggala, Monalisa Biswas, Leslie Edward S. Lewis, Vijetha Shenoy Belle, Arjun Asok, Varashree Bolar Suryakanth

**Affiliations:** 1 Department of Paediatrics, Kasturba Medical College, Manipal, Manipal Academy of Higher Education, Manipal, Karnataka 576104, India; moggala.y@learner.manipal.edu (Y.M.); leslie.lewis@manipal.edu (L.E.S.L.); 2 Department of Biochemistry, Kasturba Medical College, Manipal, Manipal Academy of Higher Education, Manipal, Karnataka 576104, India; monalisa.b@manipal.edu (M.B.); vijetha.shenoy@manipal.edu (V.S.B.)

**Keywords:** fatty acid, fatty acid methyl esters, GCMS, whole blood, plasma, serum

## Abstract

**Background/Objectives**: Fatty acids, the building blocks of lipids, contribute to numerous crucial life processes and are implicated in numerous disease pathologies. Circulating fatty acids can be extracted/trans-esterified to their respective methyl ester forms and quantified from a variety of biological samples. This study aims to identify quantifiable fatty acids (through alkali trans-esterification) in human circulation, assess the correlation of the detectable fatty acid methyl esters (FAMEs) compounds between whole blood, serum and plasma matrices and propose the most ideal matrix for quantification of FAMEs. **Methods**: This anonymised study was carried out in a tertiary hospital after obtaining ethical approval and involved analysis of residual fasting whole blood, serum and plasma samples obtained from 20 apparently healthy subjects attending the routine health check services at the study centre. Fatty acids were converted to its methyl ester form by methanolic KOH trans-esterification and subjected to GCMS analysis. Paired *t* test, Pearsons’s correlation, linear regression and Bland Altman test were employed to assess the agreeability between matrices. **Results:** 9 out of 37 FAME compounds were detected in all three matrices. Strong correlations and statistically significant regression equations were obtained for the 9 compounds between plasma and serum matrices. Undecanoate, pentadecanoate, linolenate, and palmitate levels were lowest in plasma, while stearate, heptadecanoate levels were highest in whole blood. Myristate was highest in serum, dodecanoate was highest in plasma while docosahexanoate was found to be comparable in all three matrices. Methyl ester forms of dodeconate, myristate, pentadecanoate, palmitate, heptadecanoate, stearate, and linolenate were observed in higher concentrations in plasma when compared to serum. **Conclusions:** The current study shows similar & correlating FAME concentrations between serum and plasma matrix; however, whole blood FAME concentrations appear significantly different. Plasma serves as the most ideal matrix for detection and quantification of circulating fatty acids.

## 1. Introduction

Fatty acids are aliphatic carboxylic acids which form the building blocks of all classes of lipids [1]. Human diet is replete with triglycerides composed of a variety of fatty acids contributing to numerous crucial life processes [2,3]. Fatty acids which can be synthesized by the body denovo or derived from the synthesized and/or dietary fatty acids are non-essential, while fatty acids obtained only through diet are known as essential fatty acids [4]. Fatty acids are implicated in a variety of crucial physiological functions such as metabolism, membrane physiology, hormonal regulation, storage of energy, and bio signalling [3].

Phospholipids are important determinants of membrane fluidity; while saturated fatty acids decrease membrane fluidity, PUFAs (polyunsaturated fatty acids) enhance fluidity [3]. Eicosanoids are derived either from arachidonic acid or eicosapentaenoic acid and the relative proportion of the precursor fatty acids regulate the membrane functions and have been implicated in pathophysiology of various diseases [3,4,5,6]. Saturated fatty acids are implicated in acylation of membrane proteins [7]. Fatty acids are also implicated in regulation of gene expression which in turn controls denovo synthesis of other fatty acids, lipoprotein assembly, transport and disposal, insulin resistance, and numerous pathways controlling inflammation [8,9]. Fatty acids have thus been explored in a plethora of chronic inflammatory pathologies such as atherosclerosis, cancer, immune dysfunctions etc. [3,5,7,10].

Evaluation of fatty acid composition of biological samples has been intensively explored to gain insight into the contribution of altered fatty acid metabolism in chronic life style disorders as well as malnutrition spectrum disorders [3,5,7,10]. However, due to the cost intensive nature of the instrumentation and the technical expertise involved when compared to robotic clinical laboratory medicine infrastructure, assessment of fatty acids has been restricted to high end research settings. Estimation of fatty acids in circulation has showed immense potential in risk prediction, early diagnosis and stratification of severity of numerous diseases. Fatty acids have been extracted to their methyl ester form, amenable to GCMS based detection, from a variety of biological samples, including adipose tissue, blood, breast milk, plasma, serum, and various tissues [11]. The choice of samples depends on the disease being investigated and the availability of the samples [11]. However, consensus regarding the ideal matrix to be employed and the aggregability between the matrices remains to be explored. This study aims to identify quantifiable fatty acids in human circulation and explore the aggregability of the detectable FAME compounds between three matrices: whole blood, serum and plasma. We aim to re-explore the variations of FAMEs in various blood matrices and assess whether the matrices can be used interchangeably (with regression equations for key metabolites), especially in paediatric, geriatric and chemotherapy patients where recurrent sampling is cumbersome.

## 2. Materials and Methods

Participants: The present study was carried out in the Department of Biochemistry of a tertiary hospital set up in South India after prior approval from the Institutional Ethics Committee (IEC no: 133/2023). This study involved sample analysis from 20 anonymized participants of either gender between 20–50 years of age visiting the hospital for routine health check-ups, hence informed consent was waived off (consent to publish: not applicable). Adult participants attending routine health check-ups (wellness clinics) undergo a battery of clinician requested tests/a panel of tests for half yearly/annual health/wellness assessments. Since the study was anonymised, the samples were provided with unique ID at this stage and were irreversibly delinked from the patient data.

Blood samples of these participants undergoing physician directed routine blood investigations involving the whole blood, serum, and plasma matrices were included in the study. The samples were coded and irreversibly delinked to ensure anonymity. Residual samples were collected after routine investigations and analyzed the subsequent day for the circulating concentrations of fatty acids.

### Methods

Generation of calibration curve: 

Certified reference standard for 37 fatty acid methyl esters (Food Industry FAME Mix, 30 mg/mL Total in Methylene Chloride, 1 mL/ampule, Catalogue No. 35077) was obtained from RESTEK. Serial dilution of the reference standard was carried out by using hexane as the diluent of choice to obtain a calibration curve.

Sample extraction:

300 µL of anonymised coded samples were treated with 3 mL of methanolic KOH to facilitate conversion of fatty acids to their methyl esters, the mixture was extracted with 4 mL hexane and centrifuged at 7000 rpm for 7 min. The supernatant so obtained were transferred into vials for analysis. The extraction protocol carried out is adapted from the method provided by Ichihara KI et al. [12].

GCMS parameters:

The extracted samples were analysed in gas chromatography (GC) coupled with Shimadzu Mass Spectrometer (MS), (GC/MS-QP2020 NX SHIMADZU, ShimadzuCorp., Kyoto, Japan) by selective ion monitoring method. The GC equipped with capillary column Rt-2560 (100 m, 0.25 mm ID, 0.20 µm) was used for the chromatographic analysis. The injector temperature was set at 250 °C (split mode, split ratio 4), with a column flow of 1.02 mL/min (Helium gas), and total flow is 8.1 mL/min. The oven temperature programme was set at an initial temperature of 40 °C held for 2 min, raised to 240 °C at the rate of 4 °C/min with a hold time 15 min, and a total run time of 67 min. The ion source temperature was set to 200 °C and the interface temperature was set to 250 °C.

Statistical analysis:

The concordance of fatty acid methyl esters between matrices were evaluated employing paired *t* test. Pearsons’s correlation was used to assess the correlation of fatty acids between the three matrices. Linear regression was carried out to generate a regression equation for FAMEs between matrices.

## 3. Results

**Figure 1:**
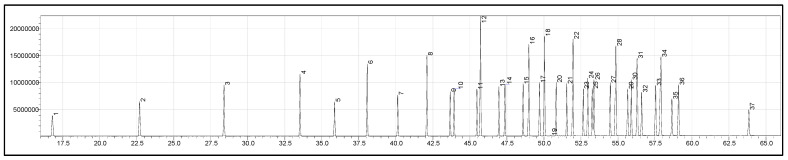
Representative chromatogram of RESTEK Food Industry FAME Mix.

The RESTEK Food Industry FAME Mix of 37 compounds was analysed in scan mode to obtain the *m*/*z* of the constituent analytes in the standard mixture. Figure 1 shows the representative chromatogram of the Food Industry FAME Mix.

Figure 1 shows the chromatogram of neat RESTEK standard in scan mode; X axis depicts the retention time and Y axis depicts the peak intensity. The numbered peaks are annotated as follows: 1. Methyl butyrate (C4:0), 2. Methyl caproate (C6:0), 3. Methyl caprylate (C8:0), 4. Methyl decanoate (C10:0), 5. Methyl undecanoate (C11:0), 6. Methyl dodecanoate (C12:0), 7. Methyl tridecanoate (C13:0), 8. Methyl myristate (C14:0), 9. Methyl myristoleate (C14:1 [cis-9]), 10. Methyl pentadecanoate (C15:0), 11. Methyl pentadecenoate (C15:1 [cis-10]), 12. Methyl palmitate (C16:0), 13. Methyl palmitoleate (C16:1 [cis-9]), 14. Methyl heptadecanoate (C17:0), 15. Methyl heptadecenoate (C17:1 [cis-10]), 16. Methyl stearate (C18:0), 17. Methyl octadecenoate (C18:1 [trans-9]), 18. Methyl oleate (C18:1 [cis-9]), 19. Methyl linoleaidate (C18:2 [trans-9,12]), 20. Methyl linoleate (C18:2 [cis-9,12]), 21. Methyl arachidate (C20:0), 22. Methyl linolenate (C18:3 [cis-6,9,12]), 23. Methyl eicosenoate (C20:1 [cis-11]), 24. Methyl linolenate (C18:3 [cis-9,12,15]), 25. Methyl heneicosanoate (C21:0), 26. Methyl eicosadienoate (C20:2 [cis-11,14]), 27. Methyl behenate (C22:0 FAME), 28. Methyl eicosatrienoate (C20:3 [cis-8,11,14]), 29. Methyl erucate (C22:1 [cis-13]), 30. Methyl eicosatrienoate (C20:3 [cis-11,14,17]), 31. Methyl arachidonate (C20:4 [cis-5,8,11,14]), 32. Methyl tricosanoate (C23:0), 33. Methyl docosadienoate (C22:2 [cis-13,16]), 34. Methyl lignocerate (C24:0), 35. Methyl cis 5,8,11,14,17-eicosapentaenoate, 36. Methyl nervonate (C24:1 [cis-15]), 37. Methyl docosahexaenoate (cis-4,7,10,13,16,19).

Following this, a selective ion monitoring method was generated (target ions and reference ions for the individual standards provided in Table 1).

**Figure 2:**
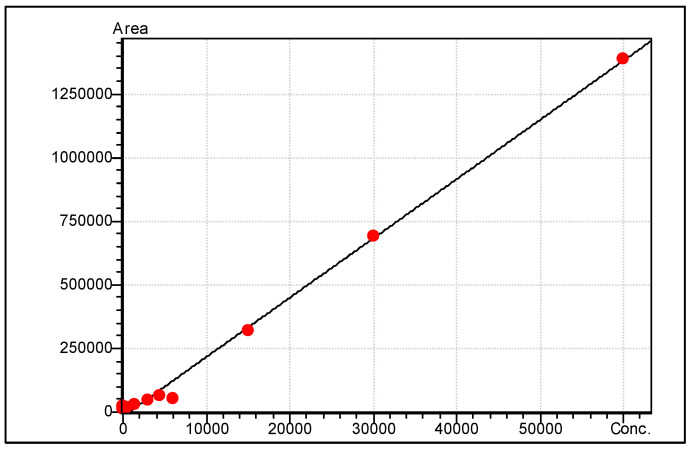
Calibration curve for methyl palmitate.

The commercial standard was serial diluted to obtain 11-point calibration curve. Figure 2 shows a representative calibration curve of methyl palmitate.

Commercial standard of methyl palmitate demonstrated linearity within the range of 1500–60,000 ng/mL. Satisfactory calibration curves were obtained for 16 FAME standards (Table 2).

Out of the above 16 FAME compounds which demonstrated adequate linearity, methyl esters of undecanoate, dodecanoate, myristate, pentadecanoate, palmitate, heptadecanoate, stearate, linolenate, eicosatrienoate and docosahexaenoate were detected in all three matrices. However, methyl eicosatrienoate was not observed at detectable ranges (with the linearity range) in all samples, hence this compound was excluded from further analysis.

The remaining 9 FAME compounds detected in all the samples were included for further analysis. Table 3 shows the comparison of the above FAME compounds between plasma and serum as well as between plasma and whole blood matrix.

Plasma matrix showed higher concentrations of most fatty acids esterified to FAMEs when compared to serum. This could be explained by the consensus that fatty acids are usually bound to plasma proteins and transported in circulation, albumin being the chief plasma protein aiding transport.

Further, correlation of FAME compounds between matrices were analysed. Table 4 shows the Pearsons correlation of fatty acid methyl esters between the three matrices.

**Figure 3:**
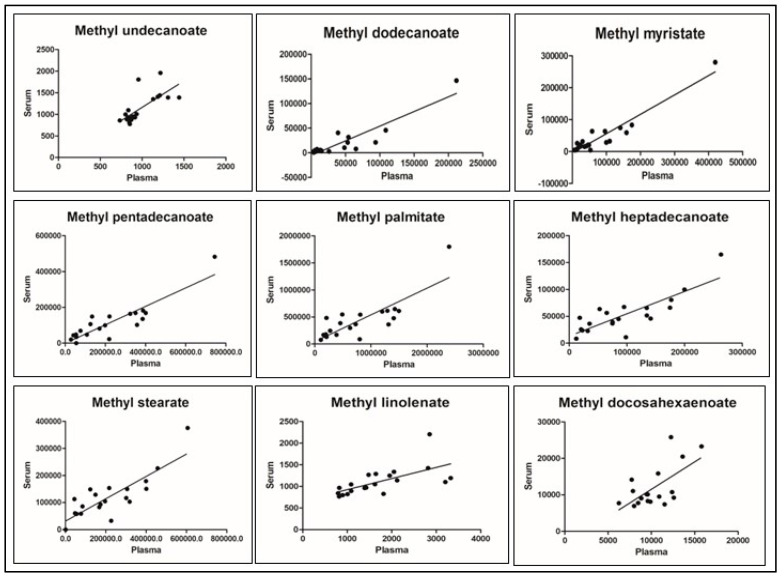
Correlation of fatty acid methyl esters between plasma and serum matrix.

Strong positive statistically significant correlation was observed for a majority of FAME compounds between plasma and serum matrix (Table 2 and Figure 3), however whole blood FAME concentrations were considerably different. Whole blood composed of 45% cellular fraction accounts for limited volume of plasma (thereby limited concentration of plasma proteins) in the 300 uL net sample volume, since majority of the fatty acids are bound to plasma proteins to aid circulation, whole blood fraction shows lower concentrations of most fatty acids and a non-significant correlation with plasma and serum matrices.

Further, regression equation was generated between matrices. Table 5 shows the Pearsons correlation of fatty acid methyl esters between the three matrices. The present data strongly suggests that plasma serves as the most ideal matrix for quantification of circulating fatty acids (post alkali transesterification to their respective methyl ester forms), however, in absence of plasma, serum might be utilized as an interchangeable matrix for quantification, with incorporation of the below regression equation.

**Figure 4:**
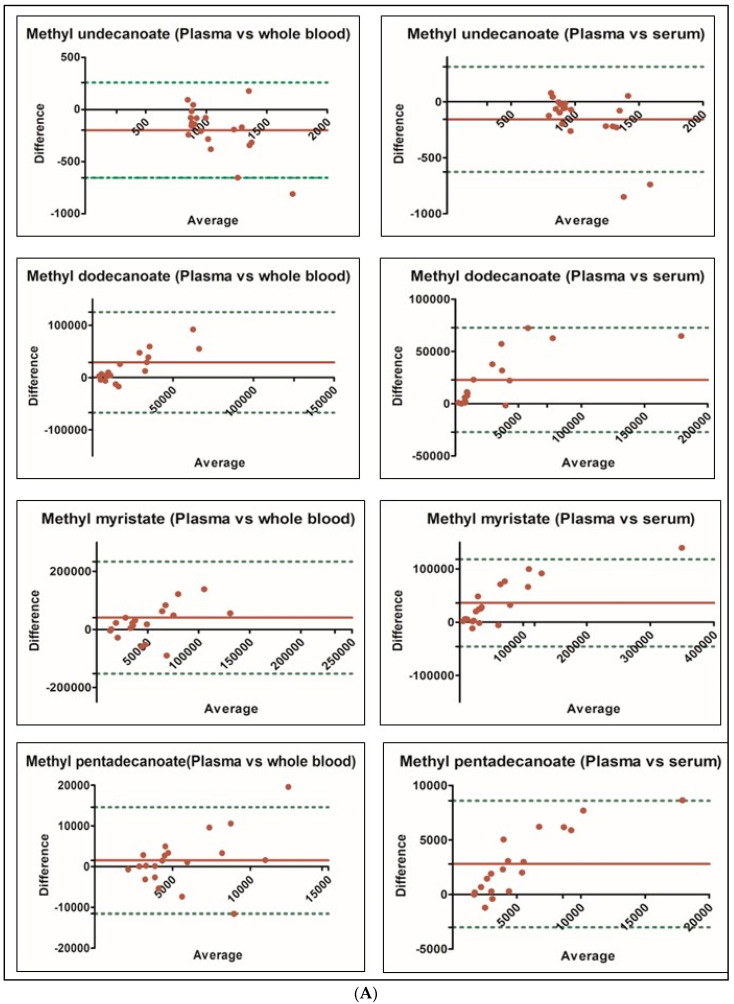

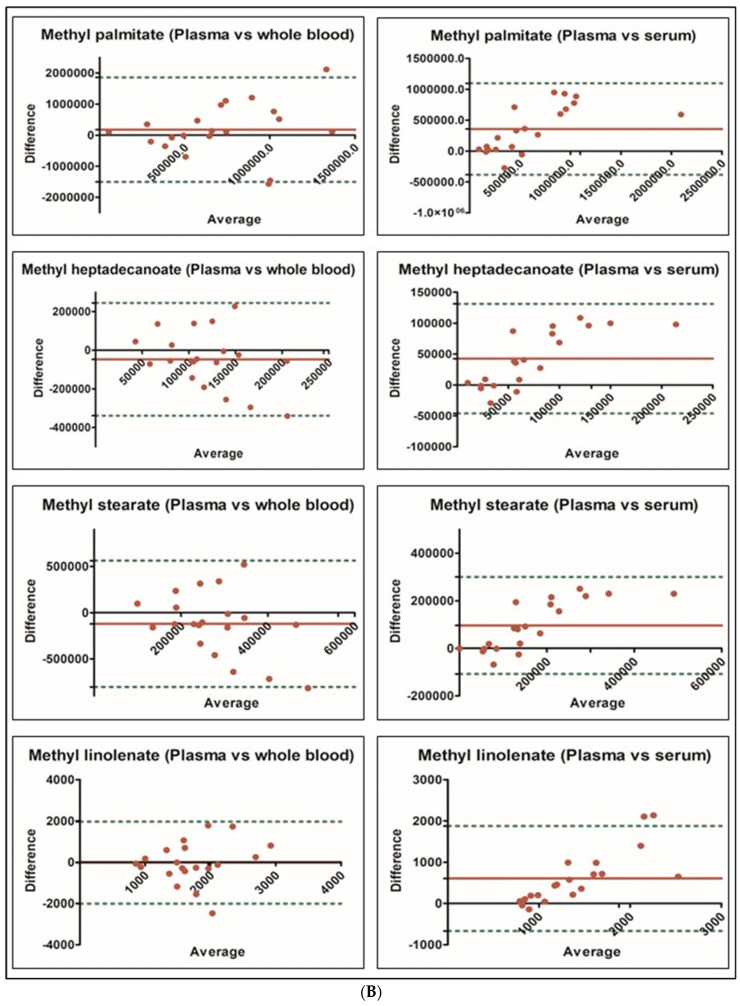

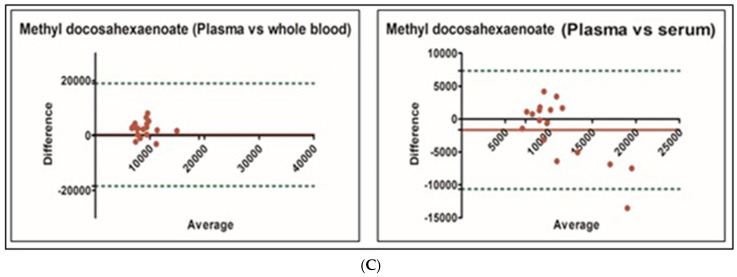
(**A**) Bland-Altman plot for methyl esters of undecanoate, dodecanoate, myristate, pentadecanoate. (**B**) Bland-Altman plot for methyl esters of palmitate, heptadecanoate, stearate, linolenate. (**C**) Bland-Altman plot for methyl esters of docosahexaenoate.

Bland-Altman plot was generated to assess the degree of agreement in the concentration of fatty acid methyl esters between the three different matrices (Figure 4A–C).

The above Bland-Altman plots show high agreement of FAME compounds within matrices, the agreement being stronger between serum and plasma matrix, when compared to whole blood. This could be attributed to the limited contribution of fluid component of the blood (plasma and plasma proteins) in the whole blood matrix (as stated above). Further, serum constitutes the fluid portion of the plasma devoid of plasma proteins, which may explain the lower abundance/concentration of major fatty acids in serum.

## 4. Discussion

Quantifying fatty acids in biological matrices is of prime importance in numerous chronic and acute lifestyle diseases where lipid metabolism or inflammation forms the underlying pathophysiology [3,5,7,10]. Circulating fatty acids, trans esterified to their respective methyl ester forms (amenable to chromatography-based separation and detection), can serve as biomarkers for various metabolic disorders, such as diabetes and cardiovascular disease [3,5,7,10]. This study explored the variations in concentration of fatty acids (derivatised as FAMEs) between various blood matrices. Out of 37 FAME compounds, detectable concentrations of only 9 FAMEs were observed in all three matrices. All assessed and quantified FAMEs showed strong statistically significant correlations between plasma and serum matrices; statistically significant regression equations were obtained to predict the concentration of assessed FAME compounds within the two matrices. Methyl pentadecanoate, methyl linolenate, methyl myristate, methyl palmitate, docosahexaenoate and dodecanoate did not show statistically significant correlations between plasma and whole blood or serum and whole blood matrix; this could probably be explained by the fact that the specified fatty acids are chief components of red blood cell membrane [13,14,15,16]. Significant correlation between all three matrices were observed only in levels of methyl undecanoate, methyl stearate and methyl heptadecanoate. Methyl undecanoate, methyl pentadecanoate, and methyl linolenate was found to be lowest in plasma, while serum and whole blood levels were comparable and did not show any significant difference. Methyl stearate and methyl heptadecanoate levels were highest in whole blood, followed by plasma with serum recording the lowest levels, the differences being statistically significant. Methyl palmitate was lowest in serum (statistically significant), while plasma and whole blood had comparable levels with higher level reported in plasma matrix. Methyl myristate was found to be highest in serum (statistically significant), while plasma and whole blood showed comparable levels. Methyl docosahexanoate was found to be comparable in all three matrices without any statistically significant differences, while methyl dodecanoate was observed to be highest in plasma, followed by serum, while lowest levels were observed in whole blood, all differences being statistically significant. Further, dodeconate, myristate, pentadecanoate, palmitate, heptadecanoate, stearate, and linolenate were observed in higher concentrations in plasma when compared to serum.

Fatty acids can be extracted in methyl ester forms, from a variety of biological samples, including adipose tissue, blood, breast milk, plasma, serum, and various tissues. The choice of sample depends on the disease being investigated and the availability of the samples. Serum, plasma and whole blood are common testing matrices, serum being the most common matrix of choice. Plasma is the liquid component of blood that contains clotting factors, while serum constitutes plasma devoid of clotting factors, the two sister matrices however show variations in percentage distribution of specific fatty acids [13,14,15,16]. Several studies have compared the fatty acid profiles of serum and plasma. In concordance with our findings, a study reported that serum and plasma show comparable levels of total fatty acids but differed in their individual fatty acid composition. The study reported that plasma had higher levels of palmitic acid and stearic acid while levels of oleic acid and linoleic acid are higher in serum [16,17]. Methyl palmitate and methyl stearate showed similar results in our study however, methyl linolenate (C18:3 [cis-6,9,12]) also showed higher levels in plasma when compared to serum. Another study reported higher saturated fatty acids and lower PUFAs in whole blood when compared to serum [17]. The study further reported that though fatty acid profiles of serum and whole blood show correlation, the levels are not identical, which is in agreement with our findings [17]. Similar studies have compared the fatty acid profiles between whole blood and packed cells and reported higher concentrations of saturated fats but lower PUFAs in packed red blood cells (PRBC). Whole blood constitutes 45% of cellular components while fatty acids are majorly transported in circulation via plasma proteins, predominantly albumin [18,19]. Hence, plasma shows higher concentration of majority of fatty acids and may be the most ideal matrix to quantify circulating fatty acids. While the present study shows good agreement between plasma and serum matrices, serum being devoid of plasma proteins, shows lower concentrations of few fatty acids. Fatty acid composition is influenced by the dietary intake and endogenous synthesis [17]. Rangel-Huerta et al. also reported higher saturated fatty acids and lower PUFAs in plasma when compared to serum after the high-fat diet, while contrasting findings were observed after a high-carbohydrate diet [17]. To partially eliminate the effect of immediate diet on the FAME profiles, our study obtained fasting specimens for analysis.

Fatty acids can forecast the risk of metabolic disorders [3,5,7,10]. Quantifying blood fatty acid methyl ester concentrations is important in inborn errors of metabolism, as it can provide diagnostic and prognostic information for these conditions [20]. FAME analysis can help identify specific inborn errors of metabolism that are related to defects in fatty acid metabolism. Additionally, FAME profiles can also be employed to monitor the efficacy of conservative and/or pharmacological or nutraceutical interventions designed to improve metabolic health and chronicity or progression of lifestyle diseases [3,5,7,10]. Despite assessing samples from apparently healthy individuals attending routine health check-ups, the current study does not establish the normal/biological reference interval of circulating fatty acid profiles in different matrices. This is due to the mandatory requirement of a minimum of 125 healthy participants of each gender (further stratified into age groups) to establish biological reference intervals, as per the CLSI guidelines, which needs to be addressed in larger studies. Further, absence of an internal standard (IS) to account for and nullify the fluctuations in MS based detection can been considered as a limitation of the present study. An ideal radiolabelled IS would enhance the analytical validity however would result in development of a cost intensive analytical method. Hence an IS based approach was not used in the present study. To ensure quality control measures, the authors suggest analysis of known control material/calibrator or pooled serum/plasma in each batch as a quality control approach (routinely used in clinical chemistry laboratories).

The current study shows similar & correlating FAME concentrations between serum and plasma matrix; however, whole blood FAME concentrations appear significantly different when compared to plasma or serum and significant correlations were observed only in levels of methyl undecanoate, methyl stearate and methyl heptadecanoate. The study proposes regression equations to predict the FAME concentration in a particular matrix from an available matrix, which could enable interchangeable testing especially in paediatric, geriatric and chemotherapy patients where recurrent sampling is cumbersome. However, pathological states selectively altering membrane lipid chemistries can generate erroneous conclusions while applying the above regression equations. Further studies on large healthy as well as disease cohorts (common lifestyle diseases like diabetes mellitus, hypertension, atherosclerosis etc) should be done to evaluate the variations of FAME profiles between matrices and the effect of the common metabolic lifestyle disorders on FAME distribution between matrices.

## 5. Conclusions

The present study explores the inter matrix variations in fatty acid concentrations (measured as FAMEs) between three blood matrices and reports plasma to be the most ideal matrix for quantification in diagnostic setups. Serum shows close agreement with plasma matrix and maybe interchangeably used for assessment of fatty acid (regression equation to predict plasma concentrations), while whole blood shows fatty acid profile distinct from the other two matrices owing to only 55% plasma volume (plasma proteins binding fatty acids) and the distinct fatty acid composition of the cellular fractions of blood, which maybe an area of interest for studies focussing of RBC membrane fragility and haemolytic diseases.

## Figures and Tables

**Table 1:** Target and reference ions for FAME analytes: Selective Ion Monitoring Parameters employed in GCMS quantification.

FAME Analyte	Molecular Weight	Target Ion *m*/*z*	Reference Ion 1 *m*/*z*	Reference Ion 2 *m*/*z*	Similarity Index *
Methyl butyrate (C4:0)	102	74	71	79	98
Methyl caproate (C6:0)	130	74	87	99	98
Methyl caprylate (C8:0)	158	74	87	55	97
Methyl decanoate (C10:0)	186	74	87	55	97
Methyl undecanoate (C11:0)	200	74	87	55	96
Methyl dodecanoate (C12:0)	214	74	87	55	96
Methyl tridecanoate (C13:0)	228	74	87	55	97
Methyl myristate (C14:0)	242	74	87	55	96
Methyl myristoleate (C14:1 [cis-9])	240	55	74	69	96
Methyl pentadecanoate (C15:0)	256	74	87	55	96
Methyl pentadecenoate (C15:1 [cis-10])	254	55	74	69	94
Methyl palmitate (C16:0)	270	74	87	75	96
Methyl palmitoleate (C16:1 [cis-9])	268	55	69	74	96
Methyl heptadecanoate (C17:0)	284	74	87	55	97
Methyl heptadecenoate (C17:1 [cis-10])	282	55	69	97	95
Methyl stearate (C18:0)	298	74	87	75	96
Methyl octadecenoate (C18:1 [trans-9])	296	55	69	83	95
Methyl oleate (C18:1 [cis-9])	296	55	69	97	95
Methyl linoleaidate (C18:2 [trans-9,12])	312	74	87	55	95
Methyl linoleate (C18:2 [cis-9,12])	294	81	67	95	96
Methyl arachidate (C20:0)	294	81	67	95	96
Methyl linolenate (C18:3 [cis-6,9,12])	326	74	87	75	93
Methyl eicosenoate (C20:1 [cis-11])	292	79	67	80	96
Methyl linolenate (C18:3 [cis-9,12,15])	324	55	69	97	96
Methyl heneicosanoate (C21:0)	292	79	67	95	94
Methyl eicosadienoate (C20:2 [cis-11,14])	340	74	87	75	92
Methyl behenate (C22:0 FAME)	322	81	67	95	95
Methyl eicosatrienoate (C20:3 [cis-8,11,14])	354	74	87	354	95
Methyl erucate (C22:1 [cis-13])	320	79	80	67	95
Methyl eicosatrienoate (C20:3 [cis-11,14,17])	352	55	69	83	91
Methyl arachidonate (C20:4 [cis-5,8,11,14])	368	74	87	79	88
Methyl tricosanoate (C23:0)	318	79	91	80	97
Methyl docosadienoate (C22:2 [cis-13,16])	350	81	67	95	95
Methyl lignocerate (C24:0)	382	74	87	382	96
Methyl cis 5,8,11,14,17-eicosapentaenoate	316	79	91	93	98
Methyl nervonate (C24:1 [cis-15])	380	55	69	83	91
Methyl docosahexaenoate (cis-4,7,10,13,16,19)	342	74	91	67	97

* Recorded by untargeted scan of the RESTEK standard.

**Table 2:** Calibration data and linearity range.

FAME Analyte	RT (mins)	R^2^	Range of Linearity (ng/mL)	Limit of Detection (ng/mL)	S/N at Lowest Calibrator	Fatty Acid Classification
Methyl butyrate (C4:0)	16.795	0.998	10,000–40,000	2625.236	16.41	SCFA
Methyl caproate (C6:0)	22.685	0.99	2000–40,000	766.3273	6.56	SCFA
Methyl caprylate (C8:0)	28.39	0.997	1000–40,000	586.615	10.89	MCFA
Methyl decanoate (C10:0)	33.525	0.997	1000–40,000	97.74512	11.23	MCFA
Methyl undecanoate (C11:0)	38.855	0.993	2000–20,000	181.1912	2.61	MCFA
Methyl dodecanoate (C12:0)	38.065	0.997	1000–40,000	544.8326	18.83	MCFA
Methyl tridecanoate (C13:0)	40.125	0.998	1000–40,000	407.3278	16.39	LCFA
Methyl myristate (C14:0)	42.09	0.998	1000–40,000	526.8825	20.42	LCFA
Methyl pentadecanoate (C15:0)	43.935	0.998	1000–20,000	422.2703	11.7	LCFA
Methyl palmitate (C16:0)	45.72	0.995	1500–60,000	1898.594	49.13	LCFA
Methyl heptadecanoate (C17:0)	47.36	0.997	500–20,000	146.9132	7.21	LCFA
Methyl stearate (C18:0)	48.98	0.994	1000–40,000	196.9079	3.48	LCFA
Methyl linolenate (C18:3 [cis-6,9,12])	51.965	0.996	200–20,000	104.1717	12.96	LCFA
Methyl eicosatrienoate (C20:3 [cis-8,11,14])	54.855	0.997	500–20,000	102.9729	7.64	LCFA
Methyl lignocerate (C24:0)	57.905	0.997	2000–40,000	1248.4	7.59	LCFA
Methyl docosahexaenoate (cis-4,7,10,13,16,19)	63.84	0.999	500–20,000	11,453.34	18.1	LCFA

**Table 3:** Comparison of fatty acid methyl esters between matrices.

Analyte	Serum (ng/mL) (Mean ± SD) n = 20	Plasma (ng/mL) (Mean ± SD) n = 20	*p* Value *	Whole Blood (ng/mL) (Mean ± SD) n = 20	*p* Value *
**Methyl undecanoate**	1133 ± 338	976 ± 197	**0.009**	1174 ± 327	0.581
**Methyl dodecanoate**	20,816 ± 34,185	41,783 ± 51,524	**0.001**	13,877 ± 9803	**0.019**
**Methyl myristate**	43,959 ± 60,502	80,079 ± 94,666	**0.001**	39,522 ± 29,581	0.769
**Methyl pentadecanoate**	3873 ± 2798	6668 ± 5289	**<0.001**	4769 ± 334	0.413
**Methyl palmitate**	440,097 ± 372,885	798,127 ± 611,299	**<0.001**	620,542 ± 488,730	0.366
**Methyl heptadecanoate**	52,902 ± 35,047	95,470 ± 69,660	**<0.001**	142,662 ± 100,234	**0.004**
**Methyl stearate**	120,966 ± 79,896	217,303 ± 160,222	**<0.001**	338,242 ± 235,317	**0.003**
**Methyl linolenate**	1104 ± 325	1709 ± 805	**<0.001**	1734 ± 666	**0.002**
**Methyl docosahexaenoate**	11,952 ± 5712	10,236 ± 2353	0.145	9966 ± 9579	0.903

* Paired *t* test (plasma and serum; plasma and whole blood).

**Table 4:** Correlation of fatty acid methyl esters between matrices.

Analyte	Serum & Plasma		Plasma & Whole Blood		Serum & Whole Blood	
	r Value	*p* Value *	r Value	*p* Value *	r Value	*p* Value *
Methyl undecanoate	0.717	<0.001	0.711	<0.001	0.516	0.020
Methyl dodecanoate	0.910	<0.001	0.337	0.158	0.224	0.388
Methyl myristate	0.950	<0.001	0.030	0.901	0.024	0.92
Methyl pentadecanoate	0.914	<0.001	−0.168	0.478	−0.147	0.549
Methyl palmitate	0.813	<0.001	−0.207	0.381	−0.135	0.569
Methyl heptadecanoate	0.826	<0.001	−0.519	0.019	−0.469	0.037
Methyl stearate	0.830	<0.001	−0.536	0.015	−0.479	0.033
Methyl linolenate	0.638	0.002	0.059	0.806	−0.193	0.414
Methyl docosahexaenoate	0.634	0.005	0.135	0.583	0.276	0.268

* Pearson’s correlation.

**Table 5:** Regression equation between different matrices.

Analyte	Serum as Predictor of Plasma (Y = a + bx)	*p* Value *	Whole Blood as Predictor of Plasma (Y = a + bx)	*p* Value *
Methyl undecanoate	501.01 + 0.419 (Serum conc)	0.0003	472.63 + 0.428 (Whole blood conc)	0.0004
Methyl dodecanoate	14,580.37 + 1.39 (Serum conc)	<0.0001	19,127.34 + 1.71 (Whole blood conc)	0.15
Methyl myristate	14,748.35 + 1.48 (Serum conc)	<0.0001	76,320.13 + 0.095 (Whole blood conc)	0.901
Methyl pentadecanoate	46,650.13 + 1.5363 (Serum conc)	<0.0001	270,415.40 − 0.3098 (Whole blood conc)	0.405
Methyl palmitate	211,899.97 + 1.33 (Serum conc)	<0.0001	958,883.32 − 0.2591 (Whole blood conc)	0.38
Methyl heptadecanoate	8577.06 + 1.64 (Serum conc)	<0.0001	146,948.56 − 0.3608 (Whole blood conc)	0.018
Methyl stearate	16,045.88 + 1.6637 (Serum conc)	<0.001	340,736.99 − 0.3649 (Whole blood conc)	0.015
Methyl linolenate	−40.3462 + 1.58 (Serum conc)	0.002	1586.054 + 0.07 (Whole blood conc)	0.806
Methyl docosahexaenoate	7116.83 + 0.26 (Serum conc)	0.004	9906.94 + 0.03 (Whole blood conc)	0.58

* Linear regression analysis.

## Data Availability

The original data pertaining to the manuscript is available with the corresponding author and shall be provided on reasonable request.
